# Consensus molecular subtyping of colorectal carcinoma brain metastases reveals a metabolic signature associated with poor patient survival

**DOI:** 10.1002/1878-0261.13748

**Published:** 2025-01-17

**Authors:** Barnabas Irmer, Darius Wlochowitz, Carolin Krekeler, Katharina Maria Richter, Suganja Chandrabalan, Michaela Bayerlova, Alexander Wolff, Georg Lenz, Lena‐Christin Conradi, Hans‐Ulrich Schildhaus, Christine Stadelmann, Veit Rohde, Martin Proescholdt, Gabriela Salinas, Kia Homayounfar, Tanja Kuhlmann, Stephan Hailfinger, Tobias Pukrop, Kerstin Menck, Tim Beissbarth, Annalen Bleckmann

**Affiliations:** ^1^ Department of Medicine A, Hematology, Oncology and Pneumology University of Münster Germany; ^2^ West German Cancer Center University Hospital Münster Germany; ^3^ Department of Medical Bioinformatics University Medical Center Göttingen Germany; ^4^ Department of General, Visceral and Pediatric Surgery University Medical Center Göttingen Germany; ^5^ Department of Pathology University Medical Center Göttingen Germany; ^6^ Department of Neuropathology University Medical Center Göttingen Germany; ^7^ Department of Neurosurgery University Medical Center Göttingen Germany; ^8^ Department of Neurosurgery University Medical Center Regensburg Germany; ^9^ NGS Integrative Genomics Core Unit, Institute of Pathology University Medical Center Göttingen Germany; ^10^ Institute for Neuropathology University Hospital Münster Germany; ^11^ Department of Hematology and Medical Oncology University Medical Center Göttingen Germany

**Keywords:** biomarker, brain metastasis, CMS, colorectal cancer, metabolism, RNA‐sequencing

## Abstract

The transcriptomic classification of primary colorectal cancer (CRC) into distinct consensus molecular subtypes (CMSs) is a well‐described strategy for patient stratification. However, the molecular nature of CRC metastases remains poorly investigated. To this end, this study aimed to identify and compare organotropic CMS frequencies in CRC liver and brain metastases. Compared to reported CMS frequencies in primary CRC, liver metastases from CRC patients were CMS4‐enriched and CMS3‐depleted, whereas brain metastases mainly clustered as CMS3 and rarely as CMS4. Regarding overall survival rates, CMS4 was the most favorable subtype for patients with hepatic lesions, followed by CMS1 and CMS2. The survival of patients with brain metastases did not correlate with CMS. However, we identified a CMS3‐related metabolic gene signature, specifically upregulated in central nervous system (CNS)‐infiltrating CRC, as a negative prognostic marker and potential tumor progressor. In summary, subtyping of CRC metastases revealed an organotropic CMS distribution in liver and brain with impact on patient survival. CNS‐infiltrating CRC samples were enriched for CMS3 and predictive metabolic biomarkers, suggesting metabolic dysregulation of CRC cells as a prerequisite for metastatic colonization of the brain.

## Graphical abstract

Colorectal cancer (CRC) metastases in liver and brain exhibited organ‐specific frequencies of consensus molecular subtypes (CMS). In terms of overall survival, CMS4 was the most beneficial subtype for patients with CRC metastases in the liver. Brain metastases were enriched for the metabolic subtype (CMS3) and certain metabolic markers that correlated with a poor overall survival of CRC patients.

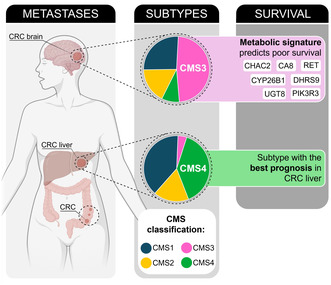

**Abbreviations**
ATRA: all‐trans retinoic acid
CMS: consensus molecular subtypes
CNS: central nervous system
CRC: colorectal cancer
dCT: delta cycle threshold
DEG: differentially expressed genes
FDR: false discovery rate
GSH: glutathione
GSVA: gene set variation analysis
HR: hazard ratios
IHC: immunohistochemistry
MSI: microsatellite instable
OS: overall survival
OXPHOS: oxidative phosphorylation
PCR: polymerase chain reaction
qRT‐PCR: quantitative real‐time PCR
RNA‐Seq: RNA‐sequencing
RSEM: RNA‐Seq by expectation–maximization
TPM: transcripts per million

## 1 Introduction

Based on global cancer statistics from 2020, colorectal cancer (CRC) is the third most frequently diagnosed tumor entity and the second leading cause of cancer‐related death worldwide [1]. In order to ensure a favorable outcome for CRC patients, treatment regimens tailored to the molecular landscape of CRC patients are pivotal. Hence, in 2015 Guinney et al. [2] established a gene expression‐based classification model to attribute primary CRC tumors to one of four consensus molecular subtypes (CMS). Each subtype was associated with certain biological features such as microsatellite instability (MSI) and immune response (CMS1), epithelial differentiation and “canonical” activation of WNT and MYC signaling (CMS2), metabolic activation (CMS3) as well as mesenchymal differentiation and stromal infiltration (CMS4).

Despite some recently proposed refinements based on single‐cell transcriptomic data [3, 4], CMS‐based clustering through bulk sequencing remains to be the “gold standard” for transcriptomic CRC patient stratification [5]. Since 2015, different studies have confirmed CMS‐specific phenotypes as predictive markers for clinical parameters such as survival and therapy response to standard‐of‐care CRC drugs [6, 7, 8, 9, 10, 11]. However, most of the existing data exclusively represent primary tumors, while neglecting metastases, which remain the predominant cause of CRC‐related death [12]. Only a few recent studies addressed the CMS classification of metastasis samples and showed CMS‐related characteristics but different subtype frequencies in hepatic lesions compared to primary tumors [8, 11, 13]. These first investigations consistently described a prognostic value for CMS classification of CRC metastases regarding overall survival (OS) of patients. Since the classification of CRC primaries alone fails to represent the heterogeneous complexity of later tumor stages [11], CMS subtyping of metastases seems to be a promising addition for improved patient stratification and can enhance our understanding of organotypic requirements for metastasis formation.

However, until now only CRC liver metastases were subjected to CMS‐based classification. Moreover, molecular drivers intrinsically mediating CMS‐dependent organotropism upon metastasis formation have not been identified yet. Next to liver, lung, and peritoneum as common destinations for CRC spreading, infiltrations of the central nervous system (CNS) are still rare with described incidences of up to 9% of all CRC patients [14]. Nonetheless, they are comparably lethal with median OS rates below 8 months [15] and increasingly detected due to improved therapies and diagnostics [16]. Thus, unraveling molecular patterns that guide CRC‐related brain tropism and negatively influence patient outcome is a matter of growing interest. Unfortunately, only little is known about biological factors that mediate tumor cell extravasation into the brain as well as cancer survival and growth within the hostile microenvironment of the CNS [17]. Furthermore, current in‐depth analyses of brain metastases mainly focus on disseminating tumors from lung, skin, and breast, while the molecular nature of CRC‐derived lesions remains uninvestigated. To address this lack of knowledge, we aimed to analyze the yet unknown molecular landscape of CRC brain metastases compared to hepatic lesions by CMS classification. Thereby, we aim to identify molecular pathways specific for CNS‐infiltrating CRC together with corresponding gene enrichments that allow for survival prediction and a better understanding of the transcriptomic requirements for CRC‐related brain colonization.

## 2 Materials and methods

### 2.1 Patients

Metastatic tissue samples were collected from a discovery cohort including 61 CRC patients, among them 38 patients with liver metastases and 23 patients with brain metastases, between July 2009 and November 2022 in Regensburg (University Medical Center Regensburg, Department of Internal Medicine III), Göttingen (University Medical Center Göttingen, Department of General, Visceral and Pediatric Surgery), and Münster (University Hospital Münster, Institute for Pathology and Neuropathology), Germany. Patient characteristics are detailed in Tables 1 and 2. Biological variables like sex and age as well as clinical parameters like tumor localization, stage, number of resected metastases, mutation, and CMS status were documented. The validation cohort comprised fresh frozen and paraffin‐embedded samples from 53 CRC patients, including 23 patients with liver and 30 patients with brain metastases (Tables S1 and S2). Due to the limited availability of human material, our validation cohort included a limited number of patients, which also had been part of the discovery cohort, that is, 2/23 for liver metastases and 9/30 for brain metastases (Tables S3 and S4). All metastasis samples were obtained after informed and written consent. The study was approved by the local ethics committees of the Medical Center Göttingen (approval no. 24/10/05 and 21/3/11) and of the Medical Association Westfalen‐Lippe (approval no. 2020‐797‐f‐S) and conducted in adherence to the standards set by the Declaration of Helsinki.

**Table 1:** Clinical parameters of patient cohort with CRC liver metastases used for RNA‐sequencing and CMS classification. Type of classification and distribution within the cohort as well as the significance level (Fisher's exact test for patient column, Wald test for trait columns) is given for each parameter. Bold and underlined *P*‐values highlight significance (*P* < 0.05). CTx, chemotherapy; F, female; LM, liver metastases; M, male; mut, mutation; *n*, number; SD, standard deviation; WT, wildtype.

Parameter	Patients				Trait, impact on OS		Trait, impact on CSS	
	Total	Pre‐treated^a^	Treatment‐naïve^a^	*P*‐value	Hazard ratio (95% CI)	*P*‐value	Hazard ratio (95% CI)	*P*‐value
Number of patients	38	23	15					
Sex M/F	24/14	15/8	9/6	1	Female: 2.23 [0.92–5.41]	0.068	Female: 2.55 [0.97–6.71]	0.05
Death during follow‐up								
Yes	16							
No	4							
Missing	18							
Mean age at metastasis resection date, years (SD)	65.73 (11.76)	63.02 (12.93)	69.89 (8.49)	0.055	≥ 65.73: 0.98 [0.4–2.39]	0.97	≥ 65.73: 0.75 [0.29–1.97]	0.563
Localization of the primary tumor								
Colon, right‐sided	11	6	11	**0.033**	Reference		Reference	
Colon, left‐sided	8	2	8		0.43 [0.09–2.11]	0.296	0.51 [0.1–2.65]	0.426
Rectum	19	15	19		1.1 [0.4–2.98]	0.858	1.2 [0.4–3.53]	0.744
Differentiation of the primary tumor, according to pathological work‐up								
Moderate (G2)	27	16	11	1	Poor: 3.47 [1.37–8.8]	**0.006**	Poor: 3.21 [1.11–8.73]	**0.023**
Poor (G3)	9	5	4					
Missing	2	2	0					
Size of primary tumor, according to TNM‐classification								
T2	2	2	0	0.345	Reference		Reference	
T3	31	19	12		1.07 [0.14–8.23]	0.948	1.02 [0.13–7.95]	0.983
T4	4	1	3		0.7 [0.06–7.81]	0.772	0.39 [0.02–6.23]	0.503
Missing	1	1	0		–	–	–	–
Nodal status, according to TNM‐classification								
N0	11	3	8	**0.04**	Reference		Reference	
N1	11	9	2		1.14 [0.25–5.1]	0.866	1.12 [0.25–5]	0.886
N2	14	9	5		4.07 [1.15–14.48]	**0.03**	3.08 [0.83–11.42]	0.093
Missing	2	2	0		–		–	
Distant metastasis at first diagnosis								
No	15	6	9	0.088	Yes: 3.21 [1.06–9.68]	**0.029**	Yes: 2.57 [0.83–7.98]	0.09
Yes	22	16	6					
Missing	1	1	0					
LM characteristics								
Total count of sequenced LM^b^	46	26	20					
Total count of resected LM	41	24	17					
Mean number of LM/patient^c^	2	2.43	1.33	**0.023**				
Interval between primary tumor and LM								
Metachronous	20	7	13	**0.004**	Synchronous: 3.21 [1.06–9.68]		Synchronous: 1.72 [0.67–4.46]	
Synchronous	21	17	4			0.083		0.257
Distribution								
Unilobar	8	5	3	0.062	Reference		Reference	
Bilobar	12	10	2		0.9 [0.28–2.91]	0.867	1.01 [0.28–3.63]	0.991
Missing/other	21	9	12		0.72 [0.24–2.16]	0.557	0.75 [0.22–2.49]	0.636
Treatment of LM								
Pre‐operative therapy				–	–	–	–	–
CTx	5	5	0					
CTx + bevacizumab	8	8	0					
CTx + cetuximab	10	10	0					
Other	1	1	0					
Extent of surgery								
Minor resection	21	10	11	0.21	Major: 1.08 [0.45–2.57]		Major: 1.03 [0.41–2.63]	
Major resection	20	14	6			0.87		0.946
R0	36	20	16	0.38	R1: 1.25 [0.37–4.32]		R1: 0.9 [0.21–3.91]	
R1	5	4	1			0.709		0.887
Post‐operative CTx								
Yes	17	9	8	0.748	No: 0.75 [0.32–1.76]		No: 0.66 [0.26–1.67]	
No	24	15	9			0.501		0.379
CMS subtype				0.14				
CMS 1	2	0	2	(0.09)	Reference	0.701	Reference	0.636
CMS 2	6	5	1		0.64 [0.07–6.22]	–	0.58 [0.06–5.63]	–
CMS 3	–	–	–		–	0.479	–	0.253
CMS 4	16	11	5		0.46 [0.05–3.93]	–	0.27 [0.03–2.54]	–
Missing	22	10	12		‐		‐	
Mutational assessment								
RAS					RAS WT: 4.37 [1.8–10.61]		RAS WT: 5.73 [2.09–15.72]	
WT	24	14	10	0.77		**0.0004**		**0.0002**
mut	21	11	10					
BRAF					BRAF WT: 0.33 [0.1–1.13]		BRAF WT: 0.29 [0.09–1.02]	
WT	43	25	18	0.57		0.064		**0.041**
mut	3	1	2					
PIK3CA					PIK3CA WT: 1.56 [0.46–5.25]		PIK3CA WT: 1.32 [0.39–4.5]	
WT	36	23	13	0.2		0.468		0.653
mut	6	2	4					
Pathological work‐up								
Necrosis, median level, in % (range)	10 (0–80)	10 (0–70)	10 (0–80)	0.53	% ≥ median: 0.7 [0.28–1.72]	0.43	% ≥ median: 0.65 [0.25–1.67]	0.37
Vital tumor, median level, in % (range)	90 (20–100)	90 (30–100)	90 (20–100)	0.53	% ≥ median: 0.85 [0.38–1.9]	0.697	% ≥ median: 0.85 [0.37–1.95]	0.698
Tumor content/sample median level, in % (range)	20 (70–90)	20 (70–90)	20 (75–90)	0.14	% ≥ median: 1.1 [0.68–3.78]	0.273	% ≥ median: 2.19 [0.85–5.66]	0.0976

^a^ Pre‐treated means that the patient received systemic cancer treatment prior to surgical resection of the liver metastases (neoadjuvant therapy). The distinct treatments are detailed in the table. Treatment‐naïve patients did not receive neoadjuvant systemic therapy.

^b^ Thirty‐eight patients were included, 41 operative procedures were performed, 46 specimen were gained and sequenced (3 patients underwent surgery at two timepoints [due to primary diagnosis and metachronous metastases], 2 patients had a resection of 5 metastases due to synchronous metastasis).

^c^ Count > 8 counted as 8.

Bold and underlined *P*‐values highlight significance (*P* < 0.05).

**Table 2:** Clinical parameters of patient cohort with CRC brain metastases used for RNA‐sequencing and CMS classification. Type of classification and distribution within the cohort as well as the significance level (Fisher's exact test for patient column, Wald test for trait columns) is given for each parameter. Bold and underlined *P*‐values are meant to highlight significance (*P* < 0.05). CTx, chemotherapy; F, female; LM, liver metastases; M, male; mut, mutation; *n*, number; SD, standard deviation; WT, wildtype.

Parameter	Patients					Trait, impact on OS	
	Total	Pre‐treated^a^	Treatment‐naïve^a^	*P*‐value	Missing treatment information	Hazard ratio (95%‐CI)	*P*‐value
Number of patients	23	4	15		4		
Sex M/F	13/10	1/3	10/5	0.262	2/2	Female: 0.83 [0.35–199]	0.679
Mean age at metastasis resection date, years (SD)	67.12 (8.0)	63.02 (12.93)	66.72 (8.39)	0.947		≥ 67.12 0.93 [0.39–3.09]	0.664
Karnofsky Index (min, median, max)	40, 70, 90	50, 70, 80	40, 70, 80	**4.82e‐08**	70,80,90	Score ≥ median 1.33 [0.47–3.73]	0.592
Localization of the primary tumor							
Colon	8	2	4	0.557	2	Reference	
Rectum	15	2	11		2	1.23 [0.49–3.09]	0.664
Other visceral metastases							
Yes	21	3	13	0.506	4	Yes: 0.18 [0.04–0.97]	**0.025**
No	2	1	2		0		
Interval primary tumor to metastasis > 2 months							
Yes	19	3	13	0.53	3	Yes: 1.02 [0.29–3.57]	0.974
No	3	1	2		0		
Missing	1	0	0		1		
Size of primary tumor, according to TNM‐classification							
T2	4	0	4	0.05	0	Reference	
T3	7	2	5		0	0.94 [0.25–3.52]	0.92
T4	2	2	0		0	3.43 [0.53–22.15]	0.19
Missing	10	0	6		4	–	–
Nodal status, according to TNM‐classification							
N0	7	2	5	0.168	0	Reference	
N1	4	0	4		0	0.62 [0.15–2.52]	0.5
N2	3	2	1		0	1.9 [0.44–8.12]	0.38
Missing	9	0	5		4	–	–
Distant metastasis at first diagnosis							
No	7	1	6	0.559	0	No: 0.99 [0.31–3.08]	0.98
Yes	7	3	4		0		
Missing	9	0	5		4		
Distribution of BM							
Supra	15	1	10	0.262	4	Supra 1.96 [0.75–5.1]	0.16
Infra	8	3	5		0		
Treatment of BM							
Radiotherapy							
No radiotherapy	6	2	4	0.833	0	Reference	
Pre‐operative	3	0	3		0	0.81 [0.16–4.24]	0.805
Post‐operative	11	2	7		2	1.08 [0.37–3.16]	0.855
Missing	3	0	1		2	–	–
Pre‐operative CTx							
Yes	4	1	3	1	0	Yes: 1.06 [0.3–3.75]	0.926
No	15	3	12		0		
Missing	4	0	0		4		
Post‐operative CTx							
Yes	10	3	7	0.582	0	Reference	
No	9	1	8		0	0.38 [0.14–1]	**0.042**
Missing	4	0	0		4	–	–
CMS subtype						Too few samples per group	
CMS 1	1	0	0	0.305	1		
CMS 2	3	1	2		0		
CMS 3	5	2	2		1		
CMS 4	1	0	1		0		
Missing	13	1	10		2		
Mutational assessment							
RAS							
WT	6	2	3	0.538	1	RAS WT: 0.87 [0.31–2.45]	0.793
mut	14	2	10		2	–	
Missing	3	0	2		1		
BRAF							
WT	18	4	11	1	3	BRAF WT: 0.33 [0.1–1.13]	0.839
mut	2	0	2		0	–	
Missing	3	0	2		1		
PIK3CA							
WT	18	4	13	–	1	PIK3CA WT: 1.56 [0.46–5.25]	0.145
mut	2	0	0		2		
Missing	3	0	2		1		
Pathological work‐up							
Necrosis, median level, in % (range)	40 (0–95)	35 (30–80)	40 (0–95)	0.96	40 (0–95)	% ≥ median: 0.54 [0.22–1.31]	0.167
Vital tumor, median level, in % (range)	60 (5–100)	65 (20–70)	60 (5–100)	0.96	35 (10–70)	% ≥ median: 0.85 [0.38–1.9]	0.79
Tumor content/sample median level, in % (range)	70 (20–100)	75 (20–95)	70 (30–100)	0.763	20 (75–90)	% ≥ median: 0.67 [0.26–1.71]	0.399

^a^ Pre‐treated means that the patient received systemic cancer treatment prior to surgical resection of the brain metastases (neoadjuvant therapy). The distinct treatments are detailed in the table. Treatment‐naïve patients did not receive neoadjuvant systemic therapy.

Bold and underlined *P*‐values highlight significance (*P* < 0.05).

### 2.2 RNA‐sequencing (RNA‐seq)

RNA‐seq data were obtained for 23 brain (GSE243188) and 46 liver (GSE162960) metastasis samples from the discovery cohort. For further analysis, gene‐level abundances were considered, which were previously estimated from FASTQ files after two pre‐processing steps [18]: (a) mapping sequencing reads against the Ensembl human reference genome GRCh38 using “Spliced Transcripts Alignment to a Reference” (v2.1.0a) [19], and (b) counting mapped reads using the “RNA‐Seq by Expectation‐Maximization” algorithm (RSEM [v1.2.19]) [20]. RNA‐Seq data of 16 primary CRC and matched liver metastasis samples were obtained from the GEO database (GSE50760). RSEM count data were estimated from FASTQ files as described above. For both datasets, RSEM count data were pre‐filtered to remove genes with low expression values. This filtering process retained only genes with counts per million > 1 in at least 10% of the samples using the bioconductor package edger (v3.8.6) [21]. Differential gene expression analysis was performed using edger. Negative binomial generalized linear models were fitted to the data, and *P*‐values were adjusted for multiple testing using the Benjamini–Hochberg (BH) method in the package to control the false discovery rate (FDR). Significantly differentially expressed genes (DEGs) were identified using a FDR < 0.05 and a |log fold change (logFC)| > 1.

### 2.3 CMS classification

To assign CMS, the r package cmsclassifier (v1.0.0) [2] was utilized. The *classifyCMS* function in the package was used with log_2_‐transformed transcripts per million (TPM) normalized counts as input, and the “RF” parameter was set to employ the Random Forest predictor for CMS classification. Briefly, the algorithm assigns samples to the nearest subtype based on classification probabilities, which is denoted as “RF.nearestCMS” in the output. The probabilities represent the confidence level of the CMS classification for each patient, with higher probabilities indicating more reliable classifications. Samples with classification probabilities below a certain threshold were not further analyzed based on exclusion criteria of the algorithm. If a sample was assigned to multiple CMS labels, the label with the lower numerical designation was selected as the final classification. To facilitate a comprehensive comparison of CMS predictions, the r package cmscaller (v2.0.1) [9], which uses the Nearest Template Prediction algorithm, was used alongside cmsclassifier. cmscaller was run on RSEM count data using default settings, but with the FDR threshold set to 1 to ensure that all samples were assigned a CMS subtype. While this approach included all predictions, it ensured a direct comparison with cmsclassifier results, considering the reliability of each prediction.

### 2.4 Hierarchical clustering and visualizations

The r package pheatmap (v4.2.1) was employed to generate heat maps and perform hierarchical clustering, with the addition of *Z*‐score transformation. The default settings for the clustering algorithm, which included complete linkage and Euclidean distance, were used. Alluvial plot visualization was done using the r package ggalluvial (v0.12.5). Survival curves were visualized using the r package survminer (v0.4.8).

### 2.5 Estimation of stromal and immune cells

The infiltration level of stromal and immune cells in tumor tissue was evaluated using the ESTIMATE algorithm [22], which leverages gene expression data. The algorithm computes two distinct score categories per patient sample: (a) stromal score, which correlates positively with stroma presence; (b) immune score, which correlates positively with immune cell infiltrations. To generate the ESTIMATE scores, TPM normalized counts were utilized as input and the r package immunedeconv (v2.1.0) was employed with default settings.

### 2.6 Estimating pathway activity using gene set variation analysis

Gene set variation analysis (GSVA) was employed to evaluate gene expression signatures and compute enrichment scores for various gene sets to assess the variation of pathway activity in an unsupervised manner [23]. The gene sets used represent 12 metabolic KEGG pathways and 14 CMS‐informative signatures, which are described in [2, 9], respectively. Log_2_‐transformed TPM normalized counts were analyzed using GSVA with default settings from the bioconductor package gsva (v1.44.5).

### 2.7 Quantitative real‐time PCR (qRT‐PCR)

Total RNA was extracted from CRC liver/brain metastases of the validation cohort using the RNeasy Maxi Kit (Qiagen, Hilden, Germany) and 1 μg was reversely transcribed into cDNA (iScript cDNA synthesis kit; Bio‐Rad, Hercules, CA, USA). The qRT‐PCR analysis was performed in triplicates with 10 ng of cDNA per reaction using the QIAquant cycler (Qiagen) and SYBR green detection. *PPIB* served as a housekeeping gene. Primer sequences are listed in Table S5.

### 2.8 Immunohistochemistry

For the quantification of protein expression in paraffin‐embedded CRC metastases (16 liver and 16 brain metastases from the validation cohort), immunohistochemistry (IHC) stainings were performed. Tissue samples were sliced into sections with a thickness of 4 μm, followed by an automated staining protocol using the Dako REAL Detection System (#K5001; Dako, Santa Clara, CA, USA) and the AutostainerLink 48 (Dako). Shortly, after deparaffinization and peroxidase activity blocking with 5% H_2_O_2_, the samples were exposed to the following primary antibodies: DHRS9 (#14560‐1‐AP), CA8 (#12391‐1‐AP), CYP26B1 (#21555‐1‐AP), PIK3R3 (#27035‐1‐AP), UGT8 (#17982‐1‐AP, all from Proteintech, Rosemont, IL, USA), CHAC2 (#HPA049235; Sigma‐Aldrich, St. Louis, MO, USA) and RET (#ab134100; Abcam, Cambridge, UK). Biotinylated secondary antibodies were applied and linked to streptavidin/peroxidase, followed by a final incubation with diaminobenzidine. Protein expression was quantified according to a previously reported protocol for semi‐quantitative determinations of protein expression based on IHC stainings [24] using the imagej software (v1.8.0), (LOCI, University of Wisconsin, Madison, WI, USA). For each patient sample, both antibody‐related (brown) and nuclear (blue) signals from three representative microscopic pictures (BZ‐X810 microscope; Keyence, Neu‐Isenburg, Germany) were separated by color deconvolution and subjected to certain thresholds splitting up specific and unspecific signals. The same threshold was used for all metastatic samples of one condition. In order to quantify protein expression values in dependence of cell numbers, the area fractions of signals associated with metabolic protein expression were normalized with the corresponding nuclear signal areas.

### 2.9 Pathway enrichment analysis

For the characterization of upregulated genes in CMS3‐classified metastasis samples compared to non‐CMS3 samples (logFC > 1, FDR < 0.05), pathway enrichment analysis was performed using four different databases: KEGG (2021 version), Reactome (2022 version), Bioplanet (2019 version), and Jensen Disease (2022 version). The results were compiled in one table and filtered for the most significant associations with metabolic processes.

### 2.10 Statistics

Differences regarding mRNA and protein expression in metastasis samples were tested for significance using either a one‐ or a two‐tailed Mann–Whitney test, depending on the null hypothesis. For protein expression values, single outliers were identified and excluded according to the Grubb's method (alpha = 0.05). Statistical calculations and visualizations were performed using the graphpad prism software (v8.4.2), (Massachusetts, USA). The impact of CMS classifications and gene set expression levels on patient OS was assessed by conducting Kaplan–Meier survival analysis. To assign patients with multiple samples to CMS in a one‐to‐one manner, the following criteria were applied: if a majority existed in the CMS assignments, the most frequent CMS was adopted; in the absence of a majority, the CMS with the lower numerical designation was adopted. The log‐rank test was employed to compare the survival rates between different patient cohorts. For the stratification of patients into two groups with high or low gene signature expression level, an optimal cutoff value was calculated based on the averaged expression level of the signature by utilizing the *surv_cutpoint* function from the survminer r package (v0.4.8). The r package survival (v3.3.1) was utilized with default settings to calculate hazard ratios (HR) and *P‐*values.

## 3 Results

### 3.1 CRC metastases display CMS‐specific properties similar to primary tumors

To evaluate organotropic CMS distributions in CRC metastases, 23 brain and 46 liver metastasis samples were resected for our discovery cohort from a total of 61 CRC patients (Tables 1 and 2). For patients with CRC liver, synchronous or metachronous diseases were observed in approximately half of the cases. Most patients with liver metastases were male; the mean age was 66 years, and the predominant localization of the primary tumor was the rectum. In contrast, most brain metastases had developed metachronously (19/23 cases). The cohort showed a nearly equal gender distribution, the mean age was 67 years and most primary tumors were located at the rectum. In both cohorts, pre‐operative treatment was applied to the majority of patients with chemotherapy being the most frequent treatment choice. After surgical resection, most patients with brain metastases were treated with chemotherapy and/or radiotherapy, while adjuvant therapy (i.e., chemotherapy) was applied to only 17/38 patients with liver metastases. In both cohorts, the most frequent molecular aberration was a *RAS* mutation with a frequency of 21/46 in liver and 14/23 in brain metastasis samples.

The transcriptomes of all metastasis samples were analyzed by RNA‐Seq and assigned to a specific molecular subtype using both the well‐established cmsclassifier and the more recent cmscaller (Fig. S1). Exclusion made by quality controls of the corresponding algorithm led to the final classification of 21 brain and 46 liver metastases. The results from both algorithms showed a high concordance, and the majority of samples were classified to the same subtype. Differences were mainly observed for CMS3, among which several samples were assigned to CMS1 with *CMScaller*, and for CMS1, for which some samples were classified as CMS3 or CMS4, respectively. Taken together, the largely overlapping results indicated the robustness of the subtype classification. Since cmscaller has been specifically designed for pre‐clinical models such as cell lines, organoids, and patient‐derived xenografts and cmsclassifier remains the gold standard for patient‐derived samples, which retain aspects of the tumor microenvironment, we chose to proceed with the subtype classification of the latter.

**Fig. 1:**
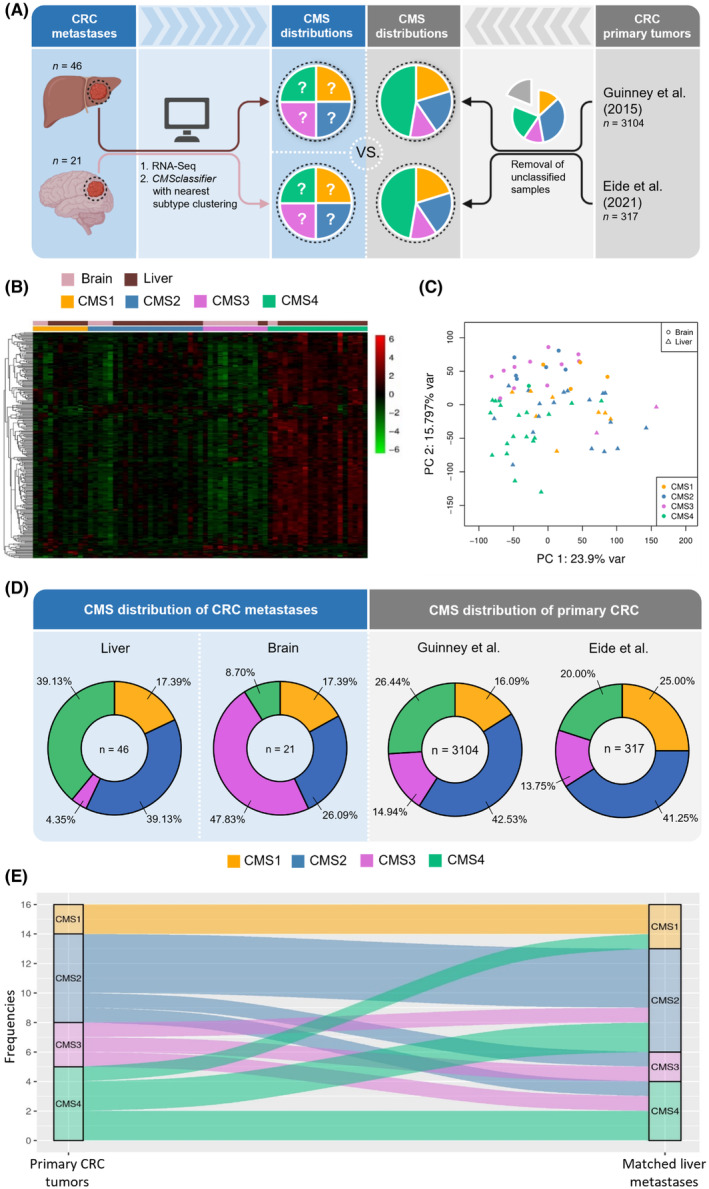
Consensus molecular subtype (CMS) metastases exhibit organotropic distributions. (A) Schematic illustration of the CMS classification workflow based on 46 hepatic and 21 brain metastasis samples resected from 59 colorectal carcinoma (CRC) patients. Samples were transcriptomically evaluated by RNA‐Sequencing with concomitant attribution of each sample to CMS 1–4. CMS classifications reported by Guinney et al. [2] and Eide et al. [11] were selected as references for primary CRC distributions. (B) Heatmap of 272 genes used by the random forest algorithm of the cmsclassifier within all resected liver and brain metastases grouped by CMS classifications. High and low gene expression are indicated by positive (red) and negative (green) *Z*‐scores, respectively. (C) Principal component clustering of CMS‐classified metastasis samples. (D) Observed CMS distributions in CRC liver and brain metastases compared to previously reported frequencies of primary CRC tumors. (E) CMS switching of primary CRC upon metastasis into the liver based on transcriptomic data (GSE50760) from 16 primary tumor samples and matched liver metastases [25].

Large cohort data published by Guinney et al. [2] and Eide et al. [11] served as references to compare the CMS distribution of primary CRC tumors and CRC brain and liver metastases (Fig. 1A). The CMS‐annotated gene expression profiles for each patient sample demonstrated subtype‐dependent expression patterns (Fig. 1B). As shown by principal component analysis, the transcriptomes assigned to CMS3 or CMS4 formed rather distinct clusters, whereas CMS1‐ and CMS2‐related gene expression patterns partially overlapped with one another (Fig. 1C). In line with previous observations from CMS classifications of primary CRC tumors [2], scoring of stromal and immune cell fractions within the respective metastatic samples via the ESTIMATE algorithm indicated relatively high infiltration rates in CMS1 and CMS4 classified samples (Fig. S2A). GSVA revealed similar CMS‐specific enrichment scores in all analyzed CRC metastases compared to previously published results of CMS‐informative signatures in primary CRC tumors [9] (Fig. S2B). Pathways with relatively increased enrichment scores comprised MSI for CMS1, microsatellite stable for CMS2, differentiation, and fatty acids for CMS3, as well as TGF‐β, epithelial‐mesenchymal transition, and LGR5 stem cells for CMS4‐classified metastases. Moreover, CMS1‐classified metastases showed the highest *BRAF* mutation frequency (16.67%) as it has been previously reported for primary CRC [2] and CRC liver metastases [11] (Fig. S2C). Unlike in the primary tumor situation, *KRAS* mutations were not only highly frequent in CMS3 metastases (58.33%) but to an even higher extent detectable in CMS1‐classified samples (66.67%) pointing towards potential metastasis‐specific alterations of *KRAS* mutation frequencies for CMS1. In combination, our results suggested that CMS‐specific attributes, initially described for primary CRC tumors, largely apply to CRC metastases.

### 3.2 CRC metastases show organotropic CMS distributions depending on tumor origin

Colorectal cancer liver metastases showed a disproportionately frequent classification as CMS4 (39.13%) compared to primary tumors (Guinney et al.: 26.44% [2]; Eide et al.: 20.00% [11]), which was accompanied by a reduced allocation to CMS3 (4.35% compared to 14.94% and 13.75%, respectively) (Fig. 1D). This observation confirmed previous studies of CRC patients with liver metastases [8, 11]. Surprisingly, however, CMS frequencies of brain metastases deviated to an even higher extent from primary tumor classification. In particular, CMS3‐classified metastases were observed to be frequently present in the brain (47.83%), representing an approximately 3‐fold increase compared to primary CRCs (Fig. 1D). In turn, CMS4 was shown to be underrepresented in brain metastases (8.70%) compared to the published classification of primary tumors. Interestingly, most of the CMS3‐classified brain metastases originated from rectal tumors, whereas colon‐derived brain metastases were majorly attributed to CMS1 (30.00%) or CMS2 (50.00%) (Fig. S3A). A correlation between the colon as primary tumor site and increased allocation of the corresponding metastases to CMS1 was similarly observed for liver metastases. Upon further assigning each metastatic sample to the left‐ or right‐sided colon as primary tumor origin, we observed higher numbers of CMS1‐classified metastases (37.50% vs. 11.76%) with a concurrent decrease in CMS2 (25.00% vs. 37.35%) and CMS4 (18.75% vs. 33.33%) for metastases derived from right‐colonic CRC compared to those originating from the left colon (Fig. S3B). This discrepancy has been consistently described earlier for primary CRCs [6] and, in our study, seemed to be mainly caused by hepatic CRC metastases. Notably, 50% of all liver metastases with a right‐colonic origin were classified as CMS1 compared to 25% of CMS1 in right‐colonic brain metastases. Taken together, we observed organotropic CMS distributions in CRC brain and liver metastases with a particular enrichment of CMS3 in the brain and CMS4 in the liver. Furthermore, rectal tumor origin was associated with increased CMS3 clustering for brain metastases, while hepatic metastases from right‐colonic tumors were strongly associated with CMS1.

### 3.3 CMS switching events as cause for organotropic CMS distributions?

Based on publicly available transcriptomic data (GSE50760) from 16 primary CRC and 16 matched liver metastasis samples [25], we addressed the question, whether intra‐patient CMS switching events during metastasis might contribute to the occurrence of organotropic CMS frequencies. After assigning each sample to one CMS, we observed remarkable differences (50% discordance) between primary and metastatic CMS allocations (Fig. 1E). We observed the highest switching rates for CMS3 (66.66%) and CMS4 (60.00%), whereas the assignment to CMS2 seemed to be more concordant between primary tumor and metastases (switching rate: 33.33%). The CMS status of both primary CRC samples classified as CMS1 remained unchanged upon metastasis, suggesting that CMS1 and CMS2 could be less prone for subtype switching. In summary, we confirmed intra‐patient CMS changes upon metastasis that suggested CMS3 and CMS4 to be the most variable subtypes, which might explain their organotropic frequencies in CRC metastases.

### 3.4 CMS3‐classified brain metastases are enriched for metabolic pathways and related metabolic genes

**Fig. 2:**
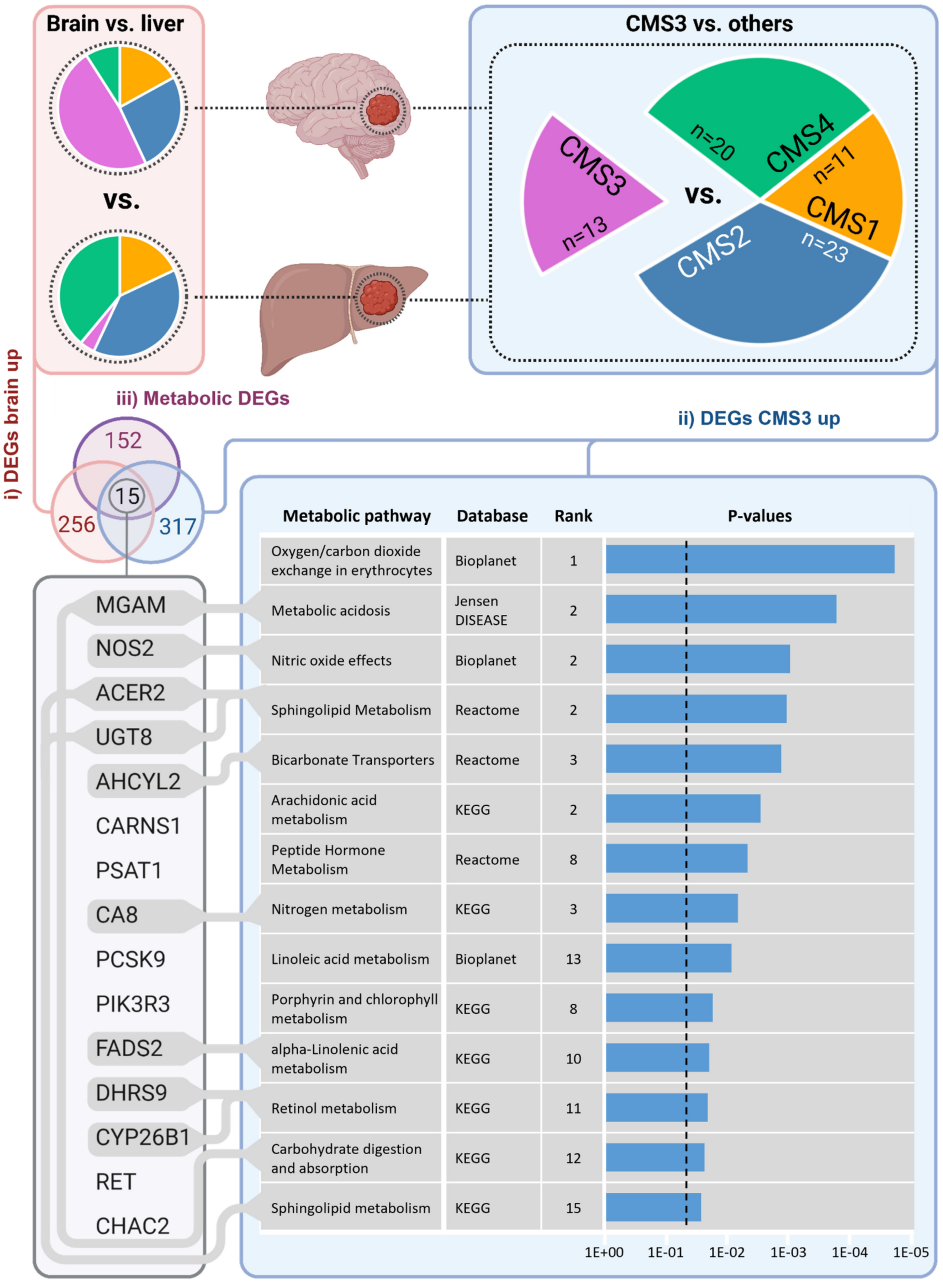
Consensus molecular subtype (CMS)3‐enriched brain metastases display an upregulation of metabolic genes. Illustration of overlapping differentially expressed genes (DEGs) that were (a) specifically upregulated in central nervous system‐resident metastases compared to hepatic lesions (logFC > 1, FDR < 0.05), (b) specifically upregulated in CMS3‐classified metastatic samples compared to all other metastases (logFC > 1, FDR < 0.05), and (c) significantly regulated in either brain or CMS3‐classified metastases (FDR < 0.05) and assigned to at least one metabolic KEGG pathway. To confirm CMS3‐related metabolic activation, pathway enrichment analysis was performed for all DEGs specifically upregulated in metastatic CMS3 (logFC > 1, FDR < 0.05). Associated pathways were ranked using four different databases (KEGG, Reactome, Bioplanet, Jensen Disease). The top 14 significantly enriched pathways linked to metabolism were listed and, if possible, assigned to certain genes of the CMS3‐ and brain‐specific metabolic signature. The vertical broken line indicates a *P*‐value of 0.05.

**Fig. 3:**
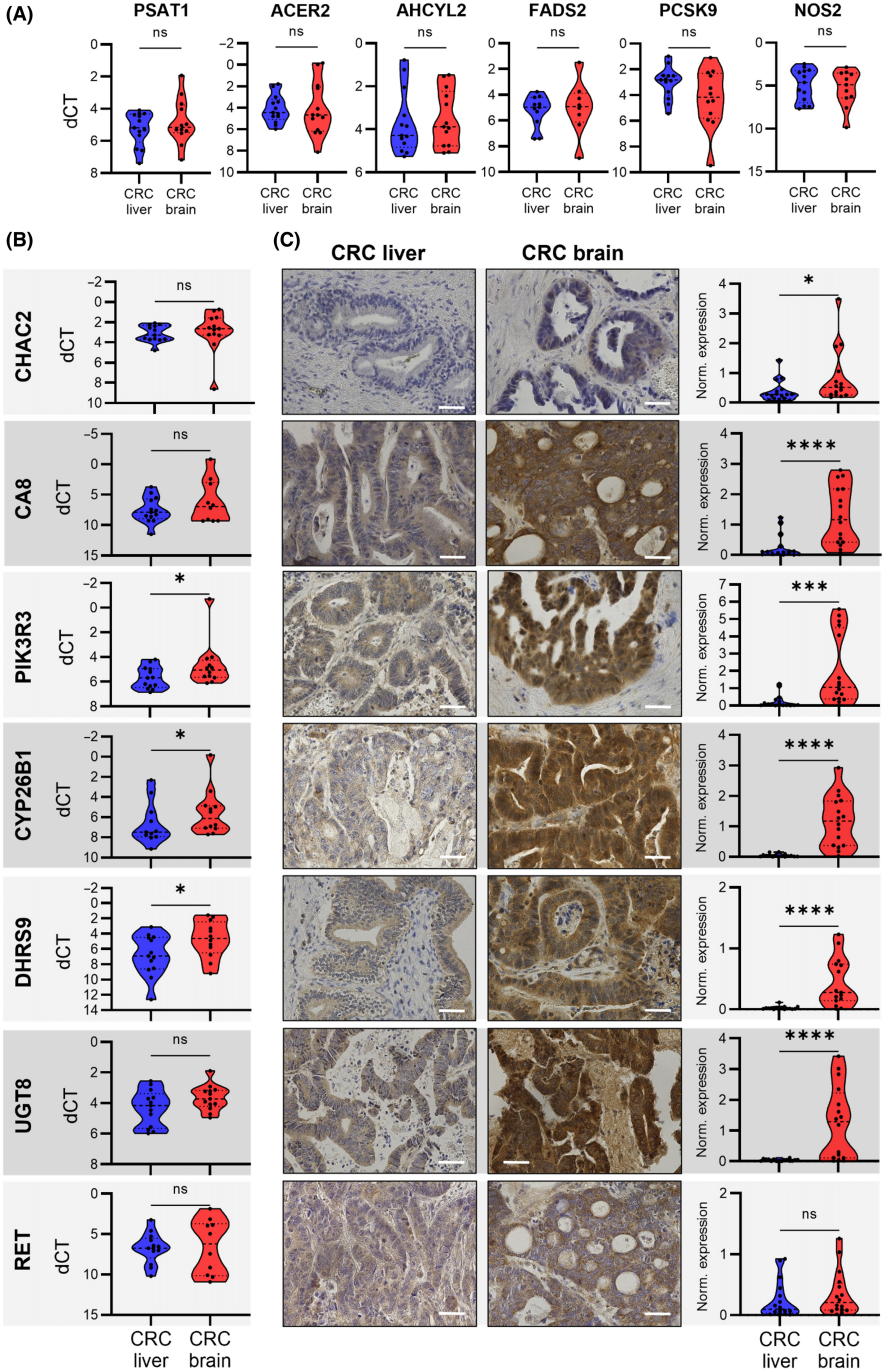
Identification of a metabolic signature with high expression levels in colorectal cancer (CRC) brain metastases. (A, B) Quantitative real‐time PCR: Expression as dCT (delta cycle threshold) values of selected metabolic genes in CRC liver and brain metastases with no evident expression differences between both metastatic entities (A) and enriched levels in brain metastases (B). Data is shown as violin plots with median as well as lower and upper quartiles. Significance was tested with a one‐tailed Mann–Whitney test (*n* = 8–13; *P < 0.05; ns, not significant). (C): Immunohistochemistry (IHC) stainings of selected metabolic genes in paraffin‐embedded samples from CRC brain and liver metastases. Shown is one representative image out of *n* = 16 liver and brain metastases. The scale bar indicates 50 μm. For the quantification of protein expression, area fractions of protein signals were normalized with respective area fractions of nuclear signals. Data is shown as violin plots with median as well as lower and upper quartiles. Significance was tested with a two‐tailed Mann–Whitney test (*n* = 16; *P < 0.05; ***P < 0.001; ****P < 0.0001; ns, not significant).

Due to the notable prevalence of CMS3 classifications in the subtype clustering of brain metastases, we aimed to further characterize CMS3‐specific features based on the transcriptomic data. Since CMS3 has been originally associated with an activation of metabolic processes in primary tumor samples [2], we strived to confirm the transferability of this observation to CRC metastases. Among the 12 metabolic KEGG pathways originally described by Guinney et al. as being upregulated in CMS3 for primary CRCs, we detected six pathways (glycerophospholipid metabolism, galactose metabolism, amino sugar and nucleotide sugar metabolism, fructose and mannose metabolism, pentose phosphate pathway, nitrogen metabolism) with relatively high enrichment scores in CMS3‐classified metastases (Fig. S4). Hence, we confirmed the activation of metabolic pathways for CMS3 in CRC metastases, though potentially differing from primary tumors with regard to their particular metabolic signature. In order to check for associations of CMS3‐classified metastases with a wider range of metabolic processes, upregulated DEGs (logFC > 1, FDR < 0.05) in CMS3‐assigned samples compared to all other subtypes were subjected to pathway enrichment analysis using four different databases (KEGG, Reactome, Bioplanet, Jensen Disease). The results were filtered for the top 14 most significant associations with metabolic processes (Fig. 2). To further define a set of metabolic genes with particularly high expression levels in CMS3‐classified brain metastases, we overlapped all upregulated CMS3‐related DEGs with the DEGs upregulated in brain compared to liver metastases (logFC > 1, FDR < 0.05). The resulting set of genes with higher expression in CMS3 and brain metastases was finally filtered for those DEGs with an annotation to metabolic KEGG pathways. As a result, we identified 15 genes, from which nine could be allocated to at least one of the most significant metabolic processes specific for CMS3 in metastatic samples (Fig. 2). Based on the transcriptomic data, we further filtered out *MGAM* and *CARNS1* due to their low expression levels in brain metastases (mean of normalized raw counts < 1). In order to confirm these findings in a largely independent validation cohort (Tables S1–S4), we measured the expression of the remaining 13 genes by qRT‐PCR in brain and liver CRC metastases. Six genes (*PSAT1*, *ACER2*, *AHCYL2*, *FADS2*, *PCSK9*, and *NOS2*) showed similar gene expression rates in brain and liver metastases (Fig. 3A). Meanwhile, *CHAC2*, *CA8*, *PIK3R3*, *CYP26B1*, *DHRS9*, *UGT8*, and *RET* were higher expressed in metastatic CRC from the brain than the liver, although the increase did not meet statistical significance for some genes (Fig. 3B). Therefore, to further validate the upregulation of the factors, we performed IHC analysis of metastasis samples from the validation cohort (Tables S1 and S2). Strikingly, the genes exhibited an even stronger brain‐specific enrichment within CRC metastases on the protein level (Fig. 3C). Only the weaker accumulation of RET protein within cerebral metastasis samples missed statistical significance. In summary, we confirmed that CMS3‐classified brain metastases display an activation of metabolic pathways. Moreover, we identified a specific set of metabolic genes specifically enriched in CRC brain metastases of the CMS3 subtype.

### 3.5 CMS in metastatic samples predict survival

**Fig. 4:**
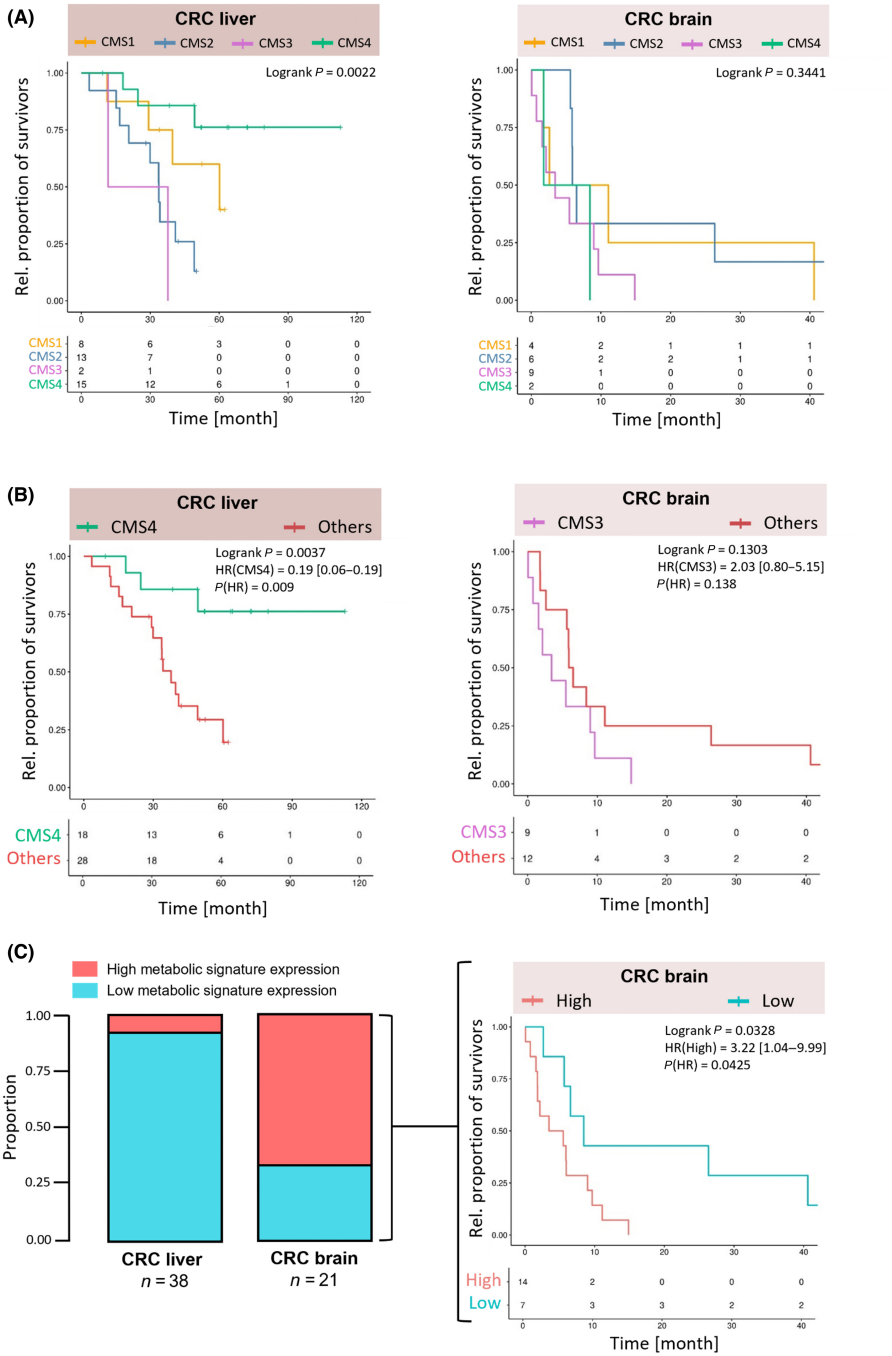
The identified metabolic gene signature is superior to consensus molecular subtype (CMS) in predicting survival of colorectal cancer (CRC) patients with brain metastasis. Overall survival (OS) of CRC patients suffering from liver or brain metastases stratified according to the CMS classification of the metastatic tissue. Survival impacts were compared between (A) all four subtypes (CMS1 vs. CMS2 vs. CMS3 vs. CMS4), (B) single subtypes and all other subtypes (CMS4 vs. others for CRC liver, CMS3 vs. others for CRC brain), and (C) high and low expression of the seven metabolic signature genes *CHAC2*, *CA8*, *PIK3R3*, *CYP26B1*, *DHRS9*, *UGT8*, and *RET* within brain metastases. An optimal cut‐off value was calculated to define high/low groups based on the transcriptomic data from metastatic samples. Survival data is shown as Kaplan–Meier curves and differences were tested with a log‐rank test. Hazard ratios with 95% confidence interval and corresponding *P*‐values are indicated for all comparisons between two cohorts.

Different studies described CMS‐dependent OS rates for patients with primary CRC [2, 6, 7] and liver metastases [8, 11]. Our data confirmed subtype‐dependent OS rates of patients suffering from CMS1‐, CMS2‐, or CMS4‐classified liver metastases (logrank *P* = 0.0022), whereas brain metastasis patients were afflicted with an overall short survival with minimal differences between the four subtypes (logrank *P* = 0.3441) (Fig. 4A). Due to the limited number of CMS3 samples in the liver and CMS4 in the brain, respectively, corresponding survival rates are likely inconclusive. CMS4 correlated with the most favorable prognosis for patients with liver metastases, followed by CMS1 and CMS2. Notably, the correlation reached statistical significance, establishing CMS4 classification of CRC liver metastases as a predictive marker for favorable CRC patient survival (HR (CMS4) = 0.1902, *P* (HR) = 0.009) (Fig. 4B). In patients with CRC brain metastases, CMS3 classification was associated with the poorest survival probabilities when compared to CMS1 and CMS2, although it did not reach statistical significance when tested as a predictive marker (HR (CMS3) = 2.026, *P* (HR) = 0.138). Nonetheless, patient stratification based on the combined gene expression of the newly defined markers for CMS3‐classified brain metastases (*CHAC2*, *CA8*, *PIK3R3*, *CYP26B1*, *DHRS9*, *UGT8*, and *RET*) was sufficient for the prediction of relevant survival differences in the discovery cohort (Fig. 4C). High expression of the metabolic marker panel within metastatic tissue samples from the brain correlated with an overall worse OS (HR (High) = 3.2244, *P* (HR) = 0.0425). In line, the same trend was observed for IHC‐based protein expression of the seven markers in the validation cohort, although it did not quite reach statistical significance when tested as a negative survival predictor (HR (high) = 2.68 [0.88–8.14], *P* (HR) = 0.082) (Fig. S5). Correspondingly, the marker genes, except *PIK3R3* and *CYP26B1*, revealed elevated transcriptional enrichment scores in at least eight different cancer entities compared to healthy tissue shown by pan‐cancer expression analysis using the TNMplot web tool [26], emphasizing their general role in cancer (Fig. S6).

## 4 Discussion

Transcriptomic classification of CRC based on CMS has been widely described for patient stratification based on primary tumor samples; however, only few studies have addressed CMS determinations for metastatic samples. Molecular subtyping of CRC metastases not only represents a promising strategy for complementing the predictive capacity of primary tumor classifications but also allows for deepening our knowledge regarding molecular prerequisites for metastatic organotropism. In this study, we comprehensively analyzed the applicability of CMS‐based classification for CRC liver and brain metastasis samples and found gene set enrichments as well as mutation frequencies comparable to primary tumor subtypes. Hence, we propose the cmsclassifier with integrated nearest subtype assignment being suitable for proper CMS classification even in metastatic settings. Using this approach, we identified a yet undescribed enrichment of the metabolic CMS3 in brain metastases. Moreover, we were able to define a panel of marker genes that was highly expressed in respective patient samples and associated with poor survival, both in the discovery as well as the validation cohort.

Consistent with data from current literature [11], we observed enriched CMS4 with concomitant decreased CMS3 classifications in hepatic lesions, whereas CMS1 and CMS2 abundances remained mostly unchanged compared to previously described CMS distributions of primary CRC tumors [2, 11]. Interestingly, high CMS1 together with low CMS2 frequencies in liver metastases were strongly associated with a right‐colonic tumor origin, whereas left colon derived metastases clustered predominantly into CMS2 with low counts for CMS1. However, due to the limited number of brain metastases, these findings require further validation in larger patient cohorts. Accordingly, metastatic liver samples from the left‐colonic rectum exhibited low CMS1 and high CMS2 abundances. A study from 2019 reported similar observations for primary CRC [6]. This suggests mainly preserved side‐of‐origin‐dependent CMS classifications upon metastasis into the liver. Opposed to that, brain metastases derived from left‐colonic rectal tumors frequently clustered into CMS3, which has not been observed yet for primary CRC or liver metastases. This might point to a side‐of‐origin‐dependent priming for organotropic subtype switches in particular for CRC brain metastasis. However, up to now, CMS switching events upon CRC invasion and organ infiltration were only reported for liver metastases [8, 11, 13] and consistently confirmed by our data. Whether CMS shifting from primary to metastatic tissue is caused by clonal expansion of single cells innately featuring the new subtype as a selection advantage in the new microenvironment or by changing gene expression patterns in metastasizing cells remains elusive. At least stromal infiltration, a potential confounder for metastatic CMS clustering, was excluded by Schlicker et al. [13] to explain the observed subtype switches. Despite yet unknown mechanisms, we suggest CMS3 and CMS4 as most susceptible to subtype switching and CMS2 as the most preserved subtype upon hepatic metastasis formation. Since matched transcriptomic data from primary tumors and corresponding brain metastases are still missing, CMS switching of CNS‐infiltrating CRC warrants further investigation.

In order to further characterize the metabolic profile of CMS3‐enriched brain metastases, we identified a metabolic gene signature, comprising *CHAC2*, *CA8*, *PIK3R3*, *CYP26B1*, *DHRS9*, *UGT8*, and *RET*, that we found specifically upregulated in CMS3‐classified tumor samples, both in the discovery as well as the validation cohort. As a hallmark of cancer, the dysregulation of metabolic pathways is a commonly known mechanism that guarantees energy supply for proliferating tumors [27]. This pivotal role of specialized cancer metabolism is not only an early‐stage phenomenon. In fact, metabolic adaptation has frequently been shown to be a prerequisite for all steps of cancer progression from primary tumor growth over invasion and circulation until extravasation and seeding into distant organs [28]. Accordingly, a study from 2019 identified organ‐specific metabolic gene expression patterns within breast cancer micrometastases [29]. This strongly suggests a causal coherence between metabolic profile and organotropism of metastasized cancer. Based on our data, we propose CRC brain tropism to be mainly linked to an overall upregulated metabolic profile of corresponding tumor cells. Consistently, previous studies reported oxidative phosphorylation (OXPHOS) and the glutathione (GSH) system being specifically upregulated in brain metastases derived from primary melanoma and breast cancer, respectively [30, 31]. These observations imply CNS‐resident metastases to depend on aerobic energy supply with GSH‐mediated protection against concomitant oxidative stress.

Interestingly, we identified *PIK3R3*, *RET*, *CA8*, and *UGT8* as enriched metabolic markers in brain metastases, which all might contribute to increased OXPHOS. *PIK3R3* induces the expression of PPARα [32], a key regulator for lipid oxidation supposed to fuel oxidative ATP synthesis [33]. *RET* is known to activate Akt signaling via its tyrosine kinase activity [34], which in turn positively regulates oxidative metabolism [35]. Akt phosphorylation was also observed for *CA8* together with impacts on glucose metabolism demonstrated by *CA8*‐related activation of aerobic glycolysis in human osteosarcoma cells [36]. Likewise, *UGT8* increased malignant proliferation through the induction of glycolytic activity in non‐small cell lung cancer cells [37]. The aforementioned reports about elevated GSH‐mediated protection against reactive oxygen species in brain metastases are in line with the organ‐specific enrichment of *CHAC2* shown by our data. Though exhibiting a catalytic activity for the slow degradation of GSH [38], *CHAC2* was shown to prevent fast *CHAC1*‐mediated GSH depletion in human embryonic stem cells, thereby facilitating GSH maintenance and oxidative stress resistance [39].

Next to increased OXPHOS and GSH stabilization, vitamin A (retinol) metabolism and its final metabolite all‐trans retinoic acid (ATRA), associated with cancer cell proliferation [40], might potentially be yet undescribed regulators for metastatic brain tropism as suggested by upregulated *DHRS9* and *CYP26B1* specific for CNS‐resident CRC metastases. By converting retinol into retinal, *DHRS9* catalyzes the first step of ATRA synthesis [41], whereas *CYP26B1* is known for its ATRA‐catabolizing activity crucial for tissue homeostasis and ATRA gradient formations [42, 43].

In addition to their potential role as effectors for metabolic adaptation of CNS‐infiltrating tumors, we demonstrated five out of seven markers to be overexpressed in at least eight different tumor entities by pan‐cancer gene expression analysis. Moreover, high metabolic marker gene expression in brain metastases correlated with an approximately three times shortened survival time compared to patients with low‐expressing tumor samples. High expression of *RET* has been already associated with poorer prognosis in other cancer entities such as neuroblastoma [44]. RET is a validated drug target, and several RET inhibitors (e.g., Selpercatinib or Pralsetinib) have been approved or are in development for RET‐driven cancers [45]. Likewise, *PIK3R3* mutations have been detected in various cancers, and overexpression has been found to correlate with advanced stage and metastasis in gastric cancer, confirming its prognostic potential [46]. The PI3K pathway, which PIK3R3 is part of, is a major drug target in oncology, and many PI3K, AKT, and mTOR inhibitors are currently tested in clinical trials. However, it is unclear yet whether PIK3R3 alterations may impact response to these inhibitors. Taken together, these results underline the impact of the newly identified metabolic gene signature on tumor progression, especially in the context of CRC brain metastasis and imply the potential use of its factors as predictive tumor biomarkers as well as novel therapeutic targets.

Of note, combined CMS3‐related gene expression levels were more suitable for survival predictions than CMS3 classification alone. Hence, with regard to patient stratification, the identification and quantification of specialized marker panels might be favored over mere CMS classification, which, at least for brain metastases, showed to be inapplicable for proper survival predictions. However, consistent with other studies, we observed CMS of hepatic lesions being correlated with patient survival in a subtype‐dependent manner. Opposing to previous results, which reported CMS2 in liver metastasis to be the most beneficial subtype followed by CMS4 and CMS1 [8, 11], our data indicated the worst prognosis for CMS2 and highest survival probabilities for CMS4. Results from Schlicker et al. [13] suggested the predictive value of CMS metastases to be influenced by the corresponding primary tumor subtype, particularly in the case of CMS2. Hence, increased numbers of unfavorable subtype switches upon primary CRC metastasis into the liver might explain the incoherently short survival rates of patients with CMS2‐classified liver metastases. Nonetheless, future investigations that address the predictive capacity of combined CMS classification, comprising both primary and metastatic tumor tissue, are still needed.

## 5 Conclusion

Based on the yet poorly characterized molecular nature of CRC‐derived metastases, we confirmed organ‐specific CMS frequencies in hepatic lesions and, to the best of our knowledge, are the first to describe organotropic CMS profiles of brain metastases. Likely influenced by subtype switching events and the primary tumor origin, CMS4 was enriched and associated with longer survival in metastatic CRC from the liver, whereas brain metastases mainly clustered into CMS3 without evident correlations between CMS status and patient survival. Taken together, our findings highlight the different cellular requirements for successful colonization of the two organs and provide valuable insight into tumor cell adaptation to the challenging brain microenvironment that can potentially serve for future predictive and therapeutic purposes.

## Conflict of interest

The authors declare no conflict of interest.

## Author contributions

BI and SC performed the *ex vivo* experiments; DW and BI performed the bioinformatic analyses; CK was involved in data acquisition and editing of the manuscript; L‐CC, H‐US, CS, VR, MP, KH, and TP were involved in the dissection, conservation and sequencing of metastasis samples; GS generated transcriptomic data; TK managed the immunohistochemical stainings of patient samples; TB, MB, and AW performed data interpretation; BI, DW, KMR, TB, SH, GL, KM, and AB analyzed the data; BI, DW, and SC wrote the manuscript; KM, TB, and AB designed and supervised the project. All authors read and approved the final version of the manuscript.

### Peer review

The peer review history for this article is available at https://www.webofscience.com/api/gateway/wos/peer‐review/10.1002/1878‐0261.13748.

## Supporting information


**Fig. S1.** CMS subtyping of CRC metastasis samples using different algorithms.
**Fig. S2.** CRC‐derived metastases in liver and brain exhibit CMS‐typical properties.
**Fig. S3.** Organotropic CMS frequencies in CRC brain and liver metastases depend on the primary tumor side.
**Fig. S4.** CMS3‐classified CRC metastases are partly enriched for metabolic CMS3‐typical gene sets.
**Fig. S5.** The metabolic gene signature shows a prognostic value also on protein level.
**Fig. S6.** Most of the metabolic genes, specific for metastatic CMS3 in the brain, are tumor‐associated and overexpressed in different cancer entities.

**Table S1.** Clinical parameters of validation cohort with CRC‐derived liver metastases used for qPCR and IHC analysis.
**Table S2.** Clinical parameters of validation cohort with CRC‐derived brain metastases used for IHC analysis.
**Table S3.** Synopsis of patients with liver metastases assessed in the validation cohort (qPCR and/or immunohistochemistry).
**Table S4.** Synopsis of patients with brain metastases assessed in the validation cohort (qPCR and/or immunohistochemistry).
**Table S5.** DNA primer used for RT‐qPCR.

## Data Availability

The transcriptomic data presented in this study are publicly available in Gene Expression Omnibus (GEO) at GSE243188 and GSE162960. Parts of the analyzed data were obtained from GEO at GSE50760. Patient data are not publicly available due to patient privacy requirements but are available upon reasonable request from the corresponding author. Raw data regarding the presented qPCR and IHC analyses are available upon request from the corresponding author.
