# Supplementary material for: Consensus molecular subtyping of colorectal carcinoma brain metastases reveals a metabolic signature associated with poor patient survival
Source: Mol Oncol. 2025 Jan 17;19(3):614–34. doi: 10.1002/1878-0261.13748 (PMC11887667; doi:10.1002/1878-0261.13748)
Supplement: Supplementary file 1 — Fig. S1. CMS subtyping of CRC metastasis samples using different algorithms. Fig. S2. CRC‐derived metastases in liver and brain exhibit CMS‐typical properties. Fig. S3. Organotropic CMS frequencies in CRC brain and liver metastases depend on the primary tumor side. Fig. S4. CMS3‐classified CRC metastases are partly enriched for metabolic CMS3‐typical gene sets. Fig. S5. The metabolic gene signature shows a prognostic value also on protein level. Fig. S6. Most of the metabolic genes, specific for metastatic CMS3 in the brain, are tumor‐associated and overexpressed in different cancer entities. [file MOL2-19-614-s001.pdf]

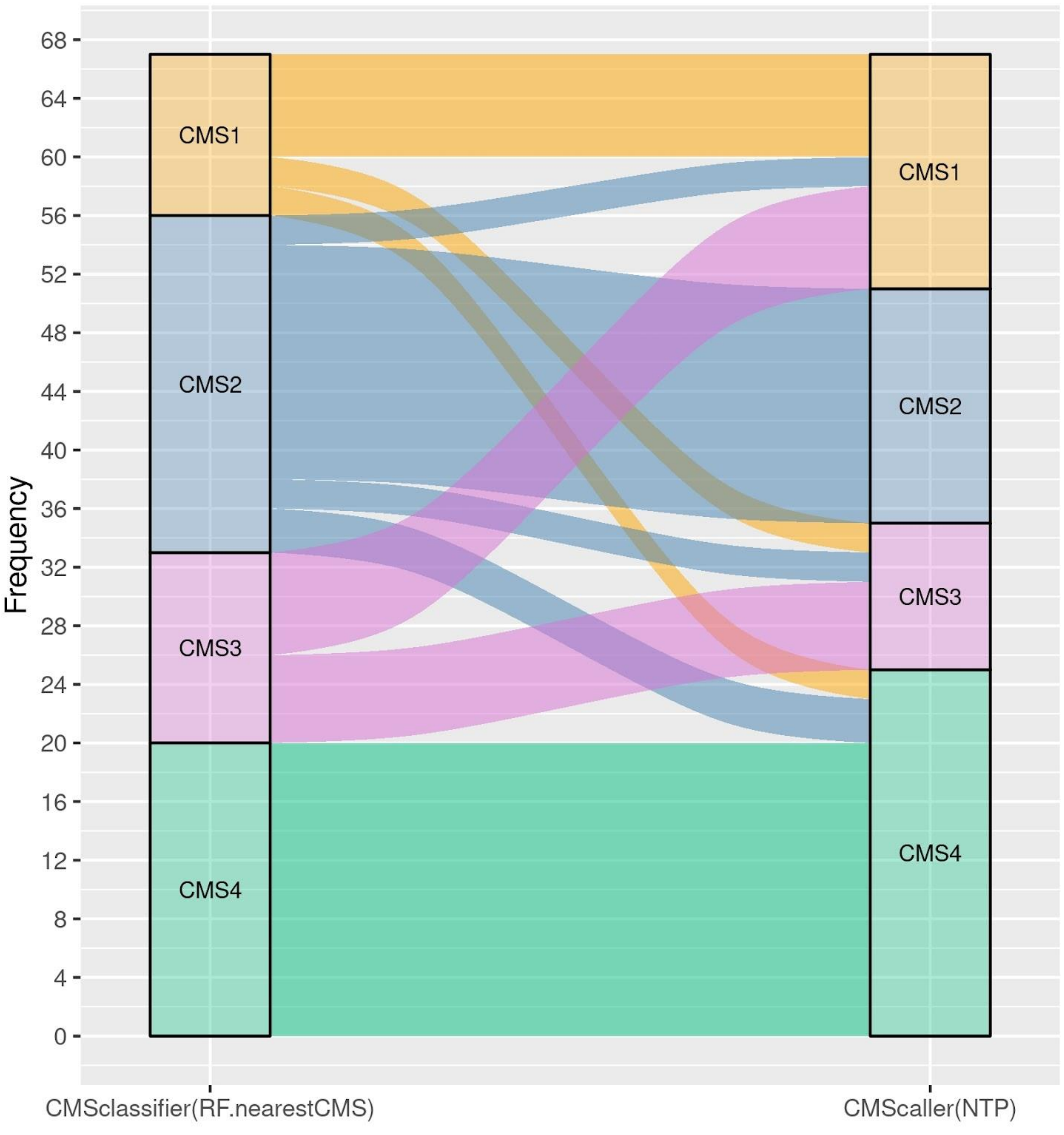

Supplementary Figure 1: CMS subtyping of CRC metastasis samples using different algorithms. Observed CMS distributions of 46 hepatic and 21 brain metastasis samples resected from 59 CRC patients. Samples were transcriptomically evaluated by RNA-Seq with concomitant attribution of each sample to CMS 1-4 by either applying the *CMSclassifier* or *CMScaller* algorithm.

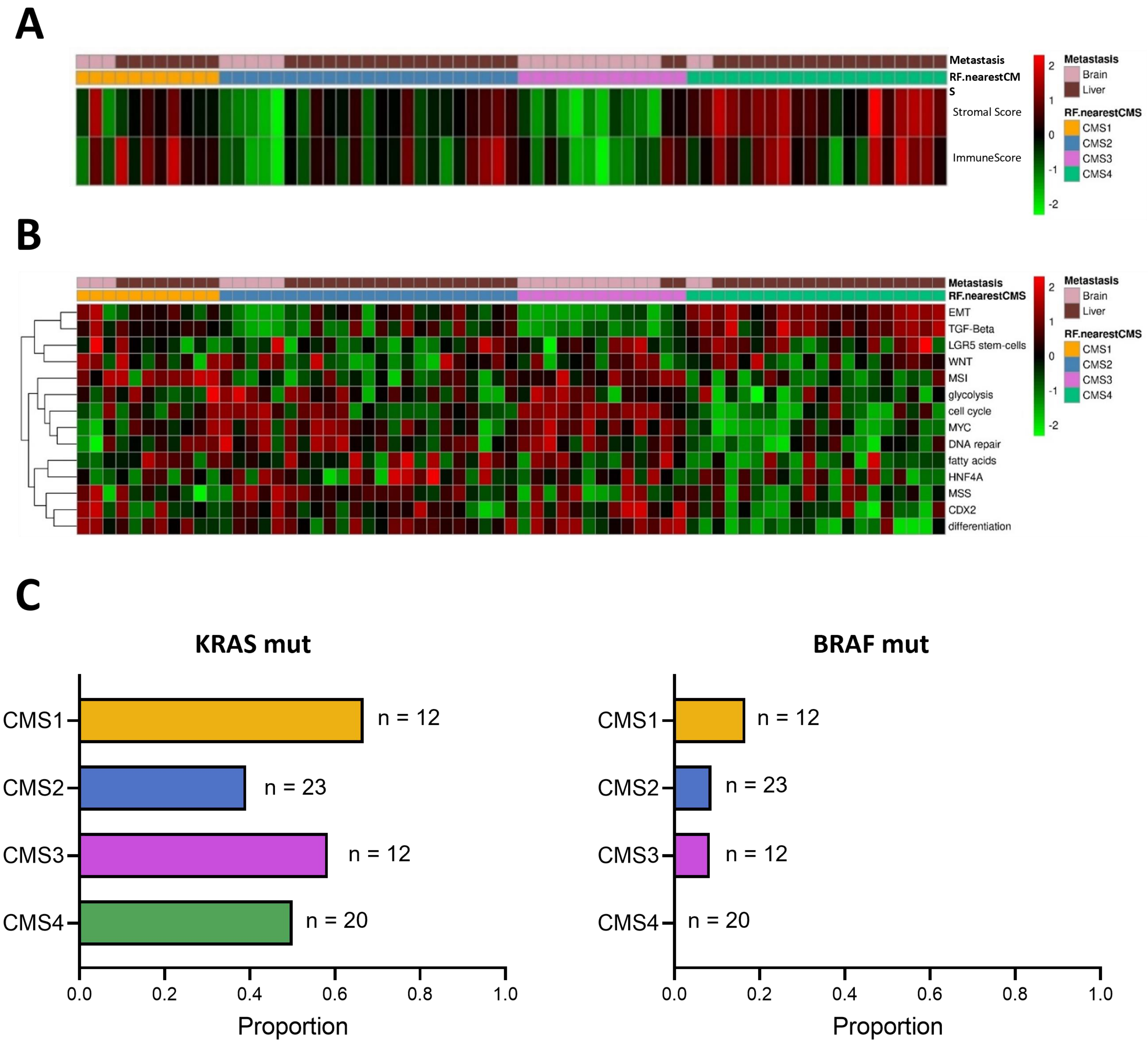

Supplementary Figure 2: CRC-derived metastases in liver and brain exhibit CMS-typical properties. A) Quantification of proposed stromal and immune cell infiltrations within all CMS-clustered samples of metastatic CRC in brain and liver based on transcriptomic data evaluations by the ESTIMATE algorithm (1). High and low infiltration rates are represented by positive (red) and negative (green) Z-scores, respectively. B) Gene set variation analysis (GSVA) of 14 previously described CMS-informative signatures (2) for all CMS-clustered samples from CRC brain and liver metastases. High and low gene set enrichments are represented by positive (red) and negative (green) Z-scores, respectively. C) Proportions of *KRAS* and *BRAF* mutations in CRC metastases in dependence of CMS.

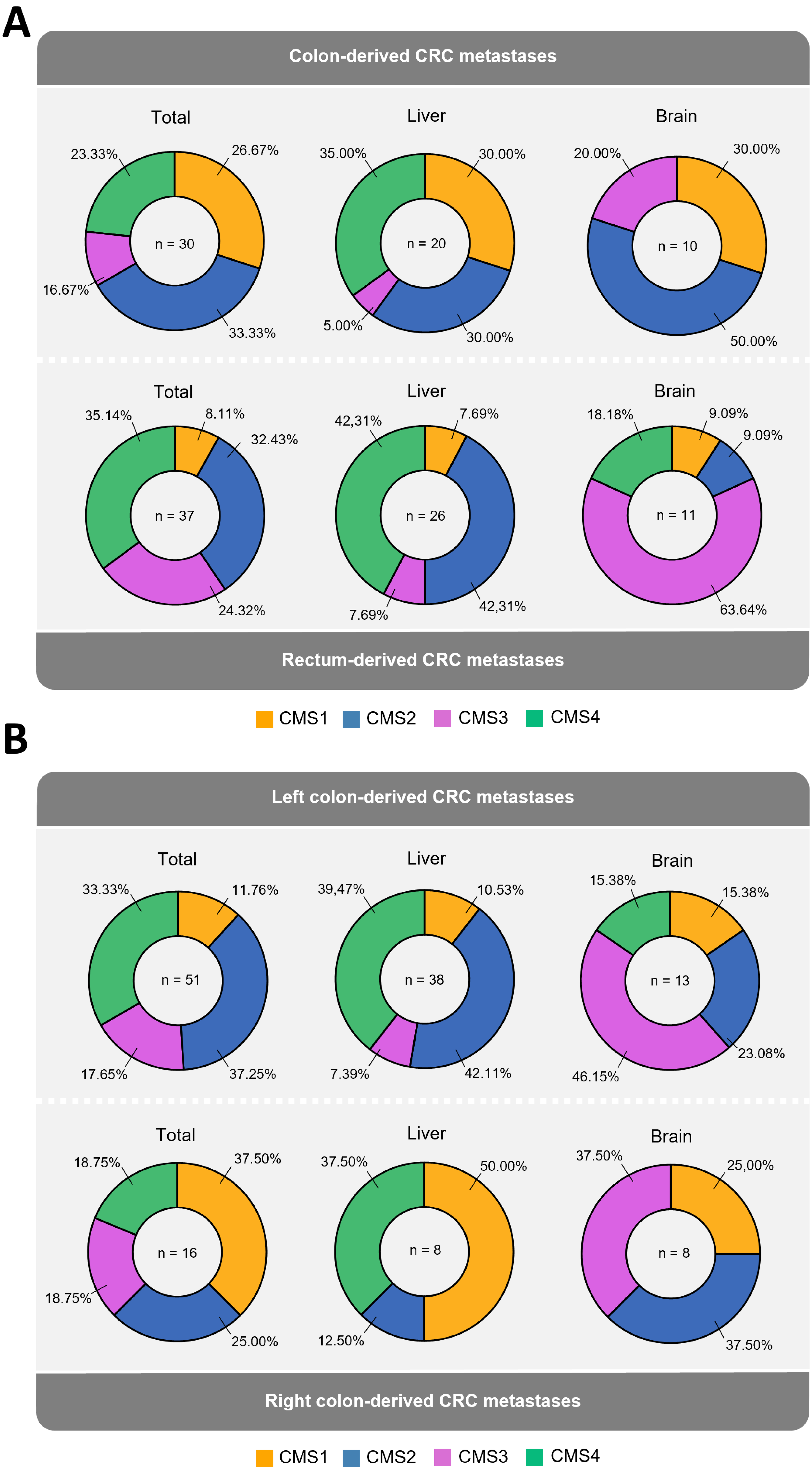

Supplementary Figure 3: Organotropic CMS frequencies in CRC brain and liver metastases depend on the primary tumor side. CMS distributions of brain (n=21) and liver (n=46) metastases originating from **A)** colon- or rectum-derived CRC and **B)** left-colonic (including rectum) or right-colonic CRC.

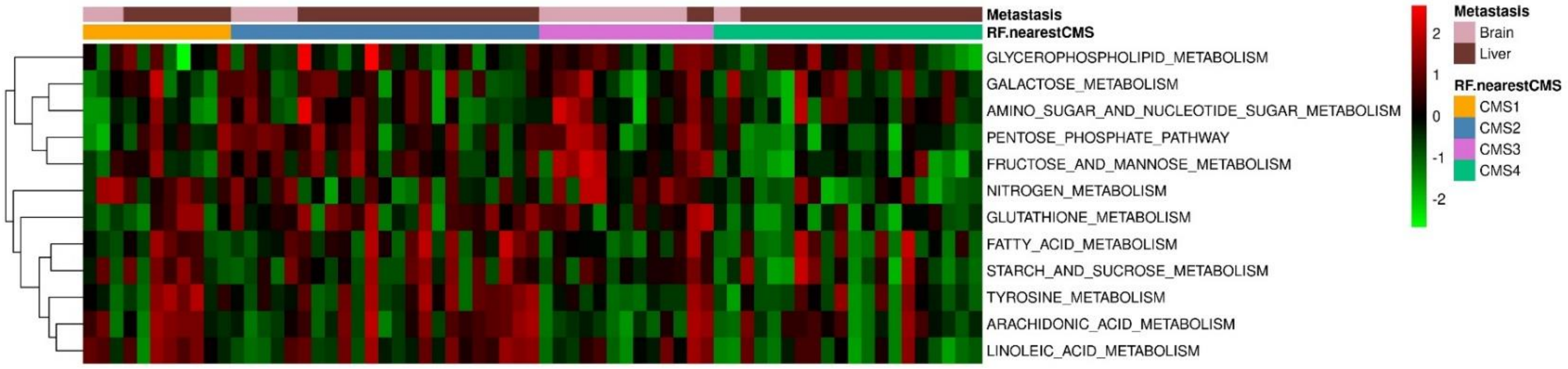

Supplementary Figure 4: CMS3-classified CRC metastases are partly enriched for metabolic CMS3-typical gene sets. Gene set variation analysis (GSVA) of metabolic KEGG pathways, previously reported as being upregulated in CMS3-classified primary CRC (3**)**, for all CMS-clustered samples from CRC brain and liver metastases. High and low gene set enrichments are represented by positive (red) and negative (green) Z-scores, respectively.

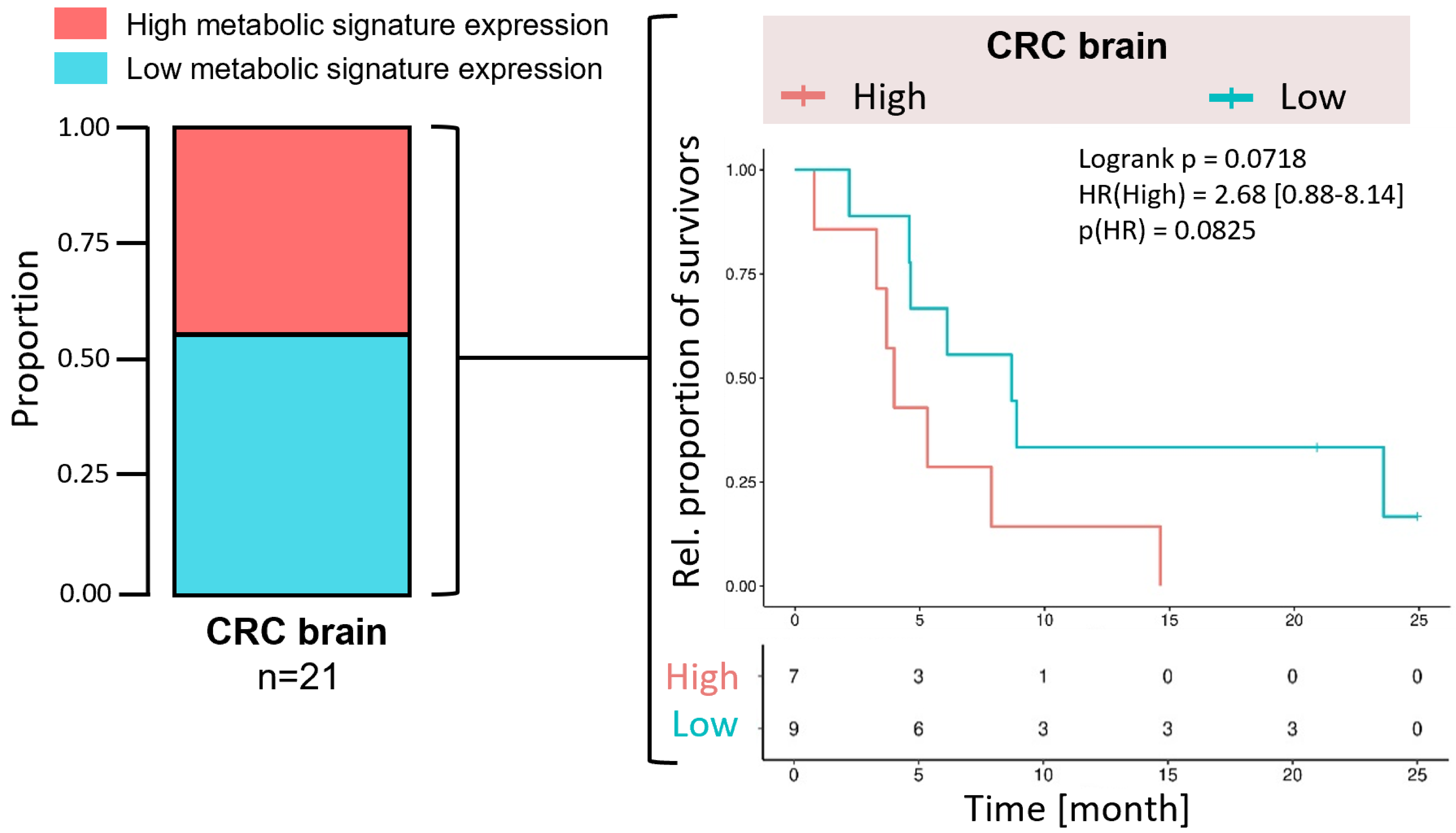

Supplementary Figure 5: The metabolic gene signature shows a prognostic value based on IHC expression data. Patients with brain metastasis were clustered into two groups according to the expression of the seven metabolic factors CHAC2, CA8, PIK3R3, CYP26B1, DHRS9, UGT8 and RET. An optimal cut-off value was calculated to define high/low groups based on quantified protein signals from the IHC analysis. OS was compared between the two groups and is shown in a Kaplan-Meier plot on the right. Differences were tested with a log-rank test. The Hazard ratio (HR) with the 95% confidence interval and the corresponding p-value are shown.

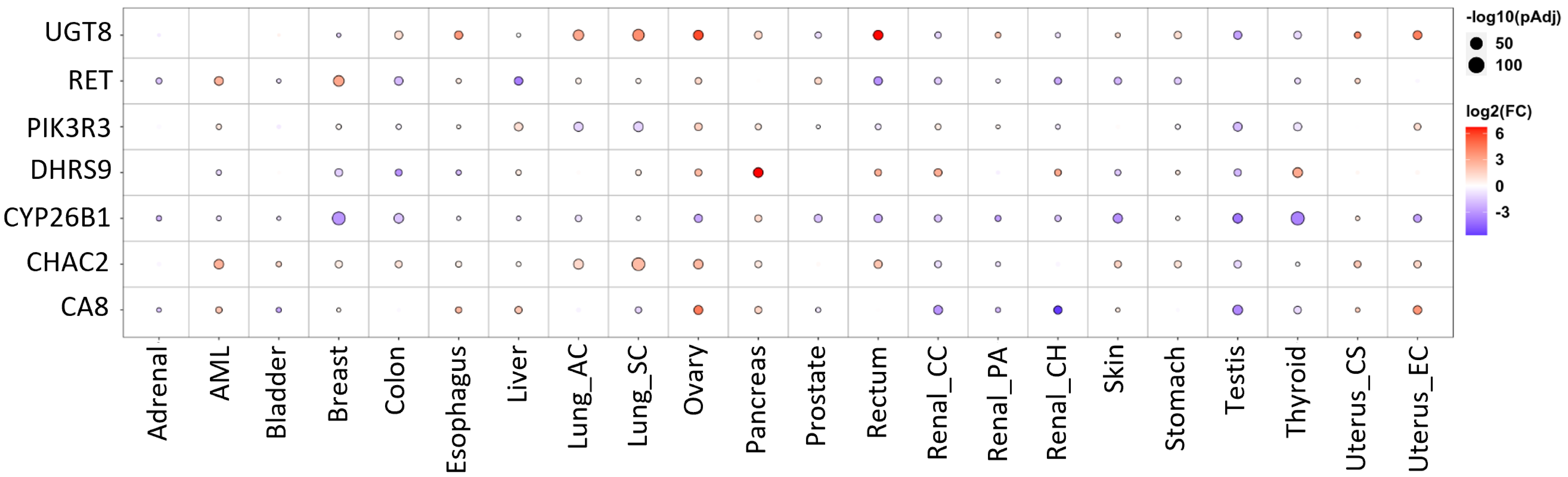

Supplementary Figure 6: Most of the metabolic genes, specific for metastatic CMS3 in the brain, are tumor-associated and overexpressed in different cancer entities. All seven CMS3-related genes that showed enriched expression in CRC brain compared to liver metastases were subjected to transcriptomic-based pan-cancer expression analysis performed by the TNMplot web tool (4). Positive (red) log2(FC) values indicate increased expression rates within cancerous tissue compared to healthy controls of the respective organ, whereas negative (blue) values represent higher expression in healthy organs. The sizes of the circles are inversely proportional to the adjusted p-value.

Supplementary References:

1. Yoshihara K, Shahmoradgoli M, Martínez E, Vegesna R, Kim H, Torres-Garcia W, et al. Inferring tumour purity and stromal and immune cell admixture from expression data. Nat Commun. 2013;4:2612.
2. Eide PW, Bruun J, Lothe RA, Sveen A. CMScaller: an R package for consensus molecular subtyping of colorectal cancer pre-clinical models. Sci Rep. 2017;7:16618.
3. Guinney J, Dienstmann R, Wang X, de Reyniès A, Schlicker A, Soneson C, et al. The consensus molecular subtypes of colorectal cancer. Nat Med. 2015;21:1350–6.
4. Bartha Á, Győrffy B. TNMplot.com: A Web Tool for the Comparison of Gene Expression in Normal, Tumor and Metastatic Tissues. Int J Mol Sci. 2021;22:2622.
