# Supplementary material for: Consensus molecular subtyping of colorectal carcinoma brain metastases reveals a metabolic signature associated with poor patient survival
Source: Mol Oncol. 2025 Jan 17;19(3):614–34. doi: 10.1002/1878-0261.13748 (PMC11887667; doi:10.1002/1878-0261.13748)
Supplement: Supplementary file 2 — Table S1. Clinical parameters of validation cohort with CRC‐derived liver metastases used for qPCR and IHC analysis. Table S2. Clinical parameters of validation cohort with CRC‐derived brain metastases used for IHC analysis. Table S3. Synopsis of patients with liver metastases assessed in the validation cohort (qPCR and/or immunohistochemistry). Table S4. Synopsis of patients with brain metastases assessed in the validation cohort (qPCR and/or immunohistochemistry). Table S5. DNA primer used for RT‐qPCR. [file MOL2-19-614-s002.pdf]

Supplementary Table 1: Clinical parameters of validation cohort with CRC-derived liver metastases.

Parameter	Patients		
	total	Pre-treated§	Treatment-naïve§
Number of patients	23	14	9
Sex M/F	14/9	7/7	8/1
Death during follow-up			
- yes	12	8	4
- no	11	6	5
Mean age at metastasis resection date, years (SD)	59.1	54.6	68.1
**Localization of the primary tumor**			
- colon, right-sided	5	3	2
- colon, left-sided	8	5	3
- rectum	10	7	3
**Differentiation of the primary tumor, according to pathological work-up**			
- good (G1)	3	2	1
- moderate (G2)	18	12	6
- *poor* (G3)	2	1	1
**Size of primary tumor, according to TNM-classification**			
- T1	1	1	0
- T2	6	4	2
- T3	14	8	6
- T4	2	2	0
**Nodal status, according to TNM -classification**			
- N0	8	4	4
- N1	10	8	2
- N2	5	3	2
**Distant metastasis at first diagnosis**			
- no	9	5	4
- yes	14	11	3
**LM characteristics**#			
- total count of resected LM	47	36	11
*- mean number of LM/patient **	3	3.3	2.75
- Interval between primary tumor and LM			
- metachronous	5	3	2
- synchronous	10	8	2
Distribution			
- unilobar	8	1	7
- bilobar	7	3	4
**Treatment of LM**			
- pre-operative therapy			
- CTx + bevacizumab	7	7	0
- CTx + panitumumab	6	6	0
- other	6	6	0
- extent of surgery			
- minor resection	13	6	7
- major resection	8	7	1
- not indicated	2	1	1
- R0	17	10	7
- R1	4	4	0
- not indicated	2	0	2
- post-operative CTx			
- yes	13	9	4
- no	9	5	4
- not indicated	1	0	1
**Mutational assessment**			
- RAS			
- WT	11	9	2
- mut	5	2	3
- not indicated	7	3	4

- *BRAF*			
- *WT*	11	8	3
- *mut*	3	1	2
- *not indicated*	9	5	4
- *PIK3CA*			
- *WT*	5	2	3
- *mut*	5	1	4
- *not indicated*	13	11	2
**Pathological work-up**			
- *necrosis, median level, in % (range)*[#]	10 (0-80)	10 (0-70)	10 (0-80)
- *vital tumor, median level, in % (range)*[#]	90 (20-100)	90 (30-100)	90 (20-100)
- *tumor content/sample median level, in % (range)*	60 (20-90)	70 (20-90)	70 (20-90)

# data only available for 15 patients

§ Pre-treated means that the patient received systemic cancer treatment prior to surgical resection of the liver metastases (neoadjuvant therapy). The distinct treatment is indicated in the table below. Treatment-naïve patients did not receive neoadjuvant systemic therapy.

Supplementary Table 2: Clinical parameters of validation cohort with CRC-derived brain metastases.

**Parameter**	**Patients**		
	total	Pre-treated§	Treatment-naïve§
Number of patients	30	10	20
Sex M/F	18/12	7/3	11/9
Mean age at metastasis resection date, years	64.8	67.7	66.45
Karnofsky Index (min, median, max)	40,70,80	50,70,70	50,70,80
**Localization of the primary tumor**			
- *colon*	14	3	11
- *rectum*	16	7	9
**Other visceral metastases**			
- *yes*	15	9	6
- *no*	10	1	9
- *not indicated*	5	0	5
**Interval primary tumor to metastasis > 2 months**			
- yes	23	6	17
- no	6	4	2
- *not indicated*	1	0	1
**Size of primary tumor, according to TNM-classification**			
- *T2*	4	3	3
- *T3*	16	6	10
- *T4*	4	2	2
- *not indicated*	6	1	5
**Nodal status, according to TNM -classification**			
- *N0*	10	1	9
- *N1*	8	4	4
- *N2*	8	4	4
- *not indicated*	3	1	2
**Distant metastasis at first diagnosis**			
- *no*	11	3	8
- *yes*	16	7	9
- *not indicated*	3	0	3
**distribution of BM**			
- supra	15	8	7
- infra	9	1	8
- both	3	1	2
- *not indicated*	3	0	3
**Treatment of BM**			
- *radiotherapy*			
- *no radiotherapy*	5	4	1
- *pre-operative*	2	2	0
- *post-operative*	17	4	13
- *not indicated*	6	0	6
- *pre-operative CTx*			
- *yes*	10	10	0
- *no*	20	0	20
- *post-operative CTx*			
- *yes*	19	7	12
- *no*	6	3	3
- *missing*	2	0	5
**Mutational assessment**			
- *RAS*			
- *WT*	4	2	2
- *mut*	10	3	7
- *not indicated*	16	5	11
- *BRAF*			
- *WT*	9	1	8
- *mut*	2	2	0
- *not indicated*	19	7	12

§ Pre-treated means that the patient received systemic cytotoxic treatment prior to surgical resection of the brain metastases in order to down-size them (neoadjuvant therapy). Treatment-naïve patients did not receive neoadjuvant systemic therapy for brain metastases prior to surgery. However, as 14/16 metastases were developed metachronously, only 2 patients were chemotherapy-naïve at the timepoint of brain surgery.

Supplementary Table 3:
Synopsis of patients with liver metastases assessed in the validation cohort.

Number	Sex	Age	Treatment prior to surgery	T	N	Metastases at first diagnosis	Stage (UICC)	Death during follow-up	Assessment in discovery cohort
1	m	56	Yes	4	1	Yes	IV	No	No
2	m	70	Yes	3	1	Yes	IV	Yes	No
3	m	65	No	3	2	Yes	IV	Yes	No
4	f	73	No	2	0	No	IV	No	No
5	f	66	Yes	3	0	Yes	IV	No	No
6	m	67	No	3	1	No	IV	Yes	No
7	f	57	Yes	3	1	Yes	IV	Yes	No
8	m	54	Yes	1	1	No	IV	Yes	No
9	m	63	Yes	2	2	Yes	IV	Yes	No
10	m	53	Yes	3	2	No	IV	Yes	No
11	m	52	Yes	2	0	No	IV	Yes	No
12	f	34	Yes	3	1	Yes	IV	No	No
13	m	57	Yes	3	1	Yes	IV	No	No
14	m	73	No	3	0	Yes	IV	No	No
15	f	61	Yes	3	0	Yes	IV	no	No
16	f	38	Yes	2	0	No	IV	No	No
17	f	72	Yes	2	0	No	IV	Yes	No
18	m	62	No	4	N+	Yes	IV	No	No
19	m	58	No	3	2b	Yes	IV	Yes	No
20	m	68	No	2	0	No	IV	No	No
21	m	68	No	3	0	No	IV	No	No
22	f	49	Yes	3	2a	Yes	IV	Yes	Yes
23	m	66	No	3b	1	Yes	IV	Yes	Yes

Supplementary Table 4:
Synopsis of patients with brain metastases assessed in the validation cohort.

Number	Sex	Age	Treatment prior to surgery	T	N	Metastases at first diagnosis	Stage (UICC)	Death during follow-up	Assessment in discovery cohort
1	m	62	Yes	4	2	Yes	IV	Yes	No
2	f	81	Yes	3	1	Yes	IV	Yes	No
3	f	60	No	n.i.	0	Yes	IV	Yes	No
4	m	60	No	3	2	No	IV	Yes	No
5	m	71	No	3	0	No	IV	No	No
6	m	58	Yes	3	0	No	IV	Yes	No
7	m	84	Yes	2	2	No	IV	Yes	No
8	m	73	Yes	3	1	Yes	IV	Yes	No
9	m	57	Yes	3	2	No	IV	Yes	No
10	m	71	No	2	0	No	IV	Yes	No
11	f	46	No	3	1	Yes	IV	n.i.	No
12	m	54	No	3	0	Yes	IV	No	No
13	f	74	No	3	2	No	IV	No	No
14	m	69	No	4	1	Yes	IV	Yes	No
15	m	68	Yes	3	1	Yes	IV	No	No
16	f	46	Yes	4	2	No	IV	No	Yes
17	f	66	No	2	0	No	IV	Yes	Yes
18	f	76	Yes	n.i.	n.i.	Yes	IV	Yes	Yes
19	f	66	No	3	0	No	IV	Yes	Yes
20	f	60	No	4a	2b	No	IV	Yes	Yes
21	m	57	No	2	0	No	IV	Yes	Yes
22	m	72	Yes	3	1	Yes	IV	Yes	Yes
23	m	69	No	3a	0	No	IV	Yes	Yes
24	f	59	No	3c	N+	No	IV	Yes	Yes
25	m	80	No	3	0	No	IV	Yes	No
26	m	76	No	n.i.	n.i.	No	IV	Yes	No
27	m	64	No	n.i.	n.i.	No	IV	Yes	No
28	f	81	No	3	1	No	IV	Yes	No
29	f	68	No	4a	2b	No	IV	Yes	No
30	m	78	No	n.i.	n.i.	No	IV	Yes	No

n.i. – not indicated

Supplementary Table 5: DNA primer used for RT-qPCR.

Gene name	Forward primer (5'-3')	Reverse primer (5'-3')
ACER2	AGTGGCATCTACTTAATCTGG	CAAGTTCATCAAGCATCTGAC
AHCYL2	GCATCGTTTGTAACATGGG	GCCAGCAGTACTATCCTC
CA8	GATGGACATACCATTCAGGT	TTGAAATTAACCGTGTGCTC
CHAC2	GTGACTCTTGTTGAAGATCC	GGCAATCTGTAAGCAACAC
CYP26B1	CCTCCTCATTGAGAGCAG	CTGCATGATGAGTGAGGT
DHRS9	TTCAATGACAGCTTAAGACG	TTACCTTTACTGGATCTGCC
FADS2	CTTCCAGATTGAGCACCA	GGCACATAGAGACTTCACC
NOS2	CAGCGGGATGACTTTCCAA	AGGCAAGATTTGGACCTGCA
PPIB	GAATTGGAGATGAAGATGTAGG	TACACGATGGAATTTGCTG
PCSK9	CCATGTCGACTACATCGAG	GGTGTCTAGGAGATACACC
PIK3R3	GTGTCCAGATACCAACAGG	TCCTGATACTGAGAGTGGT
PSAT1	GGACTATAAATATCGTTCACCC	CGCAATAATACACGTAGGAG
RET	CCGAGATGTTTATGAAGAGGA	CCATTTAACTGGAATCCGAC
UGT8	GTTATCAATAATCCCAGCTACC	TGATCCTTGTGAATTTCCGA
